# Profiles of cognitive fusion and associated factors among Chinese high school students: a latent profile analysis

**DOI:** 10.3389/fpsyg.2025.1569773

**Published:** 2025-12-01

**Authors:** Yu Ding, Ruikang Hu, Binghang Guo, Zezhong Wang, Qirui Dong, Zhifeng Zhao, Wen Wu

**Affiliations:** 1 The Battalion 2 of Cadet Brigade, School of Basic Medicine, Naval Military Medical University (NMMU), Shanghai, China; 2 Medical School of Chinese People's Liberation Army (PLA), Beijing, China; 3 The Battalion 3 of Cadet Brigade, School of Basic Medicine, Naval Military Medical University (NMMU), Shanghai, China; 4 Department of Health Management, Faculty of Military Health Service, The Navy Military Medical University (NMMU), Shanghai, China; 5 Unit 96863 of Chinese People's Liberation Army, Luoyang, China; 6 Department of Respiratory Digestive and Occupational Disease Treatment, 96608 Military Hospital of Chinese People’s Liberation Army, Hanzhong, China

**Keywords:** cognitive fusion, latent profile analysis, high school student, receiver operating characteristic area under curve, acceptance and commitment therapy

## Abstract

**Background:**

The study aimed to analyze the potential categories of cognitive fusion (CF) among Chinese high school students and to explore the cut-off values and influencing factors for distinguishing the subgroups of cognitive fusion.

**Methods:**

1,014 high school students in Hebi City, Henan Province, China were recruited by Cognitive Fusion Questionnaire from August to October 2024. Latent profile analysis was performed on the cognitive fusion score. The influencing factors associated with different classifications were investigated via multinomial logistic regression and the optimal cut-off value was identified based on the receiver operating characteristic area under curve (ROC AUC).

**Results:**

The cognitive fusion of high school students could be categorized into three subgroups: the low-CF group (14.6%), moderate-CF group (58.6%), and high-CF group (26.8%). The high-CF group identified by the questionnaire had a cut-off value of 47.5, with sensitivity at 0.996 and specificity at 0.992. For the low-CF group, the cut-off value was 30.5, sensitivity was 0.986, and specificity was 0.993. Regression analysis revealed that grade12 (aOR = 2.009, 95%CI: 1.323–3.050) and female (aOR = 1.563, 95%CI: 1.085–2.250) were linked to the moderate-CF group. Additionally, students in grade 11 (aOR = 1.940, 95% CI: 1.156–3.256), grade 12 (aOR = 1.704, 95% CI: 1.063–2.730), and females (aOR = 2.147, 95% CI: 1.426–3.233) were more likely to belong to the high-CF group.

**Conclusion:**

High school students’ cognitive fusion scores can be classified into three potential categories. The screening tool demonstrates effectiveness in identifying high cognitive fusion groups, with significant differences observed across categories based on gender and grade level. Tailored interventions targeting the specific characteristics of each category may contribute to reducing cognitive fusion among high school students.

## 1 Introduction

Cognitive fusion (CF) is one of the core pathological concepts proposed by Hayes in acceptance and commitment therapy (ACT). When individuals’ cognition is excessively governed by rigid language rules or entrenched thinking patterns, they are prone to perceive their own thoughts or emotions as absolute truths. Consequently, this mindset constrains their actions and curtails their capacity for embracing broader perspectives and adaptive behaviors. Ultimately, they are unable to respond flexibly to their actual environment. In other words, in a state of “fusion,” an individual becomes trapped within their own mental constructs, treating the content of their thoughts as literal and objective realities rather than recognizing them as transient and subjective psychological phenomena (Belisle and Dixon, 2022; Hayes et al., 2013; Hayes et al., 2006; Shao et al., 2024). More simply, fusion means that your cognition governs your behavior (McCall, 2014). Cognitive fusion, which is frequently marked by an absence of mental flexibility, plays a substantial role in the onset and maintenance of various psychological disorders (Chen et al., 2023). CF is often associated with poorer mental health outcomes, such as depression and anxiety, and has been proposed as one of the core processes that may explain the link between vulnerability and psychopathology (Cookson et al., 2020; Gillanders et al., 2014). Therefore, enhancing research on CF among high school students has emerged as a crucial direction in crisis intervention studies. Early identification of CF risks enables the implementation of timely and targeted interventions, which can effectively address the needs of different risk groups.

Latent Profile Analysis (LPA) is an individual-centered category analysis technique. It identifies homogeneous groupings or categories with similar features from multivariate data by analyzing the interactions between multiple features (Anthony and Robbins, 2013; Su et al., 2024). This approach identifies potentially homogeneous subgroups based on probability distributions (Marsh et al., 2009), and explains the relationship between indicators of external continuous variables through potential categorical variables (Hou and Zhang, 2023). In recent years, latent profiling has been widely used in psychology, medicine and other fields due to its advantages in identifying potential groups and analyzing complex data structures (Wang et al., 2017).

This study adopted the individual-centered latent profile analysis method, taking high school students in Hebi City, China as the research subjects. Based on the scores of each item of the Cognitive Fusion Questionnaire (CFQ), the potential categories of CF among Chinese high school students were determined and its influencing factors were explored. Firstly, LPA is used to analyze the potential characteristics of high school students in Hebi City, China. Secondly, the effects of demographic variables, such as gender, grade and annual household income, on different potential categories are analyzed. Finally, the ROC curve was used to determine the optimal cut-off value of the CFQ score to help classify and screen potential categories of CF. Previous studies mainly focused on the mediating effect between CF and certain mental states, but few of them explored the potential classification pattern or related factors of CF in high school students. This analysis will assist schools and parents in identifying high-risk groups of CF promptly, thereby enabling the development of effective intervention and treatment strategies to prevent more severe psychological crises. At the same time, it provides an empirical basis for the acceptance and commitment therapy to carry out psychological treatment of high school students, and provides a more accurate group classification basis for predicting other related negative psychological variables.

## 2 Methods

### 2.1 Participants

By using convenient sampling, 1,129 students from several high schools in Hebi City, China were investigated online through the Questionnaire Star platform from August to October 2024. In the process of testing, the quality control was carried out by using reverse scoring questions, balancing the order of items and guiding the real report of the subjects. Questionnaires with excessively short response times, overly regular or consistent answers, as well as those containing incorrect or missing responses, were excluded.1014 completed the survey effectively, yielding an effective response rate of 89.8%. The other 115 questionnaires were excluded because of invalid data.

The study complies with the Declaration of Helsinki and has been approved by the Institutional Ethics Committee of Hospital of Unit 96,608 of the People’s Liberation Army (PLA) of (NO. 2024-A-004), and all participants participated voluntarily and obtained their informed consent.

### 2.2 Measures

#### 2.2.1 Sociodemographic data collection

Sociodemographic data were gathered using a self-designed questionnaire. Sociodemographic variables, including gender, grade, age, place of residence, family annual income, family structure (having siblings or being an only child) and the class cadre.

#### 2.2.2 Chinese version of the cognitive fusion questionnaire (CFQ)

CFQ developed by Zhang Weichen from Institute of Psychology (Zhang et al., 2014), Chinese Academy of Sciences was adopted in this study. There were 9 items in the questionnaire, and Likert was scored by 7 points, with 1 representing “obviously inconsistent” and 7 representing “obviously consistent.” A higher score on the subject indicates a higher level of cognitive fusion. It features a single-factor structure and demonstrates internal consistency. In this study, the Cronbach’s *α* coefficient of this questionnaire was 0.95.

### 2.3 Quality control

The assessment was organized uniformly on a class-by-class basis, using a group administration method via computer networks. The experimenters are psychology professionals who have received unified training. Prior to completing the questionnaire, participants were given unified instructions by the experimenters regarding the assessment purpose, filling method and important precautions. Additionally, they were assured that their responses would be kept confidential. The questionnaires were collected and sorted out immediately after the test.

### 2.4 Statistical methods

Descriptive statistics were used to describe the variables measured in terms of frequency, percentage, mean, and SD. Mplus7.4 and SPSS27.0 software are used. Data analysis was divided into three steps: (1) Mplus 7.4 was used for LPA analysis, and the scores of each item on the CFQ were used as explicit response index variables to establish a latent profile model and estimate the fit of the latent profile model for high school students’ potential risks. The statistical indicators for evaluating the goodness of fit of the model are as follows: Akaike information criterion (AIC), Bayesian information criterion (BIC), information Entropy (Entropy), etc. The best class model is determined according to the principle that the model fits better if Entrop y is higher and AIC, BIC, and sample-size adjusted BIC (aBIC) are lower. The model classification quality indicator Entropy represents the difference between the classification accuracy and error rates of the model. Entropy ranges from 0 to 1. Entropy > 0.8 indicates that the classification accuracy reaches 90%, and the closer to 1 indicates that the classification is more accurate (Hou and Zhang, 2023). (2) The accuracy of the CFQ in this study was tested by receiver operating characteristic (ROC) curve analysis. (3) The obtained cognitive fusion risk category was taken as the dependent variable, and gender, grade, age, place of residence, family annual income, family structure and the class cadre were taken as the independent variables. The Multiple Logistic regression was carried out to further improve the recognition and differentiation accuracy of potential risk features of cognitive fusion. The statistical difference was *p* < 0.05.

## 3 Results

### 3.1 Characteristics of the participants

A total of 1,014 valid samples were collected, including 359 from Grade 10 (35.4%), 256 from Grade 11 (25.2%), and 399 from Grade 12 (39.3%). Among the participants, there were 443 males (43.7%) and 571 females (56.3%), with an average age of 16.4 ± 0.9 years (ranging from 14 to 19 years). Regarding family annual income, 840 students (82.8%) reported incomes between 0 and 100,000 yuan; 150 (14.8%) reported incomes between 100,000 and 200,000 yuan; 20 (2.0%) reported incomes between 200,000 and 300,000 yuan; and 4 (0.4%) reported incomes of ≥300,000 yuan. In terms of residential areas, 177 students (17.5%) lived in urban areas, while 641 (63.2%) resided in rural areas, and 196 (19.3%) lived in township areas. Additionally, there were 29 only children (2.9%) and 985 non-only children (97.1%). Among the participants, 194 (19.1%) were class cadre members, whereas 820 (80.9%) were not.

### 3.2 LPA model of cognitive fusion

Based on the scores from the 9 items of the CFQ, LPA was employed to identify the potential subgroups of cognitive fusion among high school students in Hebi City, China. This was achieved by successively evaluating models with 1 to 5 potential categories. AIC, BIC and aBIC all decreased gradually with the increase of the number of categories. Entropy is higher than 0.8 for two to five categories, with Entropy greater than 0.9 for three of them. LMRT reached the significant level (*p* < 0.001). LMR prompts category 3 to be optimal (Table 1). Considering the simplicity, accuracy and actual situation of the model, the model of category 3 is finally selected as the best model, and the ascription probabilities of the potential category 3 are shown in Table 2.

**Table 1:** Fitting index and class probability of latent profile analysis model of cognitive fusion.

Models	AIC	BIC	aBIC	Entropy	VLMR-LRT *p*-value	BLRT *p*-value	Categorical probability
1	33707.15	33795.74	33738.57	-	-	-	1.00
2	29863.95	30001.75	29912.82	0.876	0.0032	<0.001	0.56/0.44
3	27176.05	27363.07	27242.38	0.954	<0.001	<0.001	0.15/0.59/0.27
4	26514.22	26750.46	26598.01	0.896	0.0041	<0.001	0.37/0.34/0.10/0.19
5	26249.29	26534.75	26350.54	0.895	0.0167	<0.001	0.05/0.11/0.30/0.36/0.19

AIC, Akaike information criteria; BIC, Bayesian information criterion; aBIC, adjusted Bayesian information criterion; Entropy Classification accuracy index; BLRT, bootstrap likelihood ratio test; LMR-LRT, Lo-Mandell-Rubin likelihood ratio test.

**Table 2:** Average attribution probability of the third latent category model of cognitive fusion.

Category	Attribution probability		
	Profile 1 (*n* = 148)	Profile 2 (*n* = 594)	Profile 3 (*n* = 272)
Profile 1	0.968	0.032	0.000
Profile 2	0.007	0.982	0.011
Profile 3	0.000	0.027	0.973

In the category model, the category’s own attribution probability [that is, the average probability that high school students (rows) in each category belong to each potential category (columns)] ranges from 96.8 to 98.2%. The attribution probability (i.e., attribution error) for other categories ranges from 1.8 to 3.2%, which demonstrates the high accuracy of the three-category model employed in this study and confirms the reliability of the classification results.

**Figure 1:**
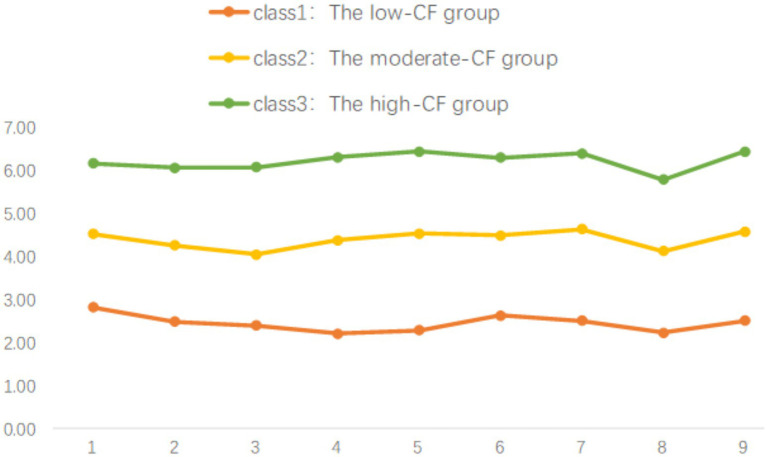
Latent profile plot based on the CF for high school students in Hebi, China (The x-axis shows Cognitive Integration Scale entries, while the y-axis represents the mean score for the 9 items of the Cognitive Fusion Questionnaire. The three lines show the scores of the 3 categories on 9 items of the CFQ).

Each category was labeled based on its distinct characteristics. Students in Category 1 achieved the lowest scores across the nine items and were designated as the “low-CF Group,” comprising 14.5% (*n* = 148) of the sample. Students in Category 2 scored in the middle range, representing 58.6% (*n* = 594) of the participants, and were labeled as the “moderate-CF Group.” Individuals in Category 3 obtained the highest scores on all items, accounting for 26.8% (*n* = 272), and were classified as the “high-CF Group.” According to the classification results of potential categories, the scores of the 3 categories on 9 items of the CFQ were plotted, as shown in Figure 1.

Univariate analysis of potential profile influencing factors of different potential categories: The three potential categories of cognitive fusion of high school students in Hebi City, China had statistically significant differences in gender and grade (*p* < 0.05) (Table 3).

**Table 3:** Differences in demographic variables among the latent classes (*n*, %).

Variables	The low-CF group (*n* = 148) (%)	The moderate-CF group (*n* = 594) (%)	The high-CF group (*n* = 272) (%)	Total (%)	*χ* ^2^	*P*
Gender					13.295	0.001
Male	81 (54.7)	263 (44.3)	99 (36.4)	443 (43.7)		
Female	67 (45.3)	331 (55.7)	173 (63.6)	571 (56.3)		
Age					17.891	0.057
14	1 (0.7)	4 (0.7)	1 (0.4)	6 (0.6)		
15	37 (25.0)	104 (17.5)	48 (17.6)	189 (18.6)		
16	54 (36.5)	197 (33.2)	93 (34.2)	344 (33.9)		
17	41 (27.7)	241 (40.6)	106 (39.0)	388 (38.3)		
18	15 (10.1)	47 (7.9)	20 (7.4)	82 (8.1)		
19	0 (0.0)	1 (0.2)	4 (1.5)	5 (0.5)		
Family annual income					9.695	0.138
RMB0-100,000	126 (85.1)	487 (82.0)	227 (83.5)	840 (82.8)		
RMB100,000–200,000	16 (10.8)	95 (16.0)	39 (14.3)	150 (14.8)		
RMB200,000–300,000	6 (4.1)	8 (1.3)	6 (2.2)	20 (2.0)		
RMB >300,000	0 (0.0)	4 (0.7)	0 (0.0)	4 (0.4)		
Address					4.330	0.363
Urban area	27 (18.2)	97 (16.3)	53 (19.5)	177 (17.5)		
Rural area	100 (67.6)	374 (63.0)	167 (61.4)	641 (63.2)		
Township area	21 (14.2)	123 (20.7)	52 (19.1)	196 (19.3)		
Grade					15.184	0.004
Grade10	70 (47.3)	197 (33.2)	92 (33.8)	359 (35.4)		
Grade11	32 (21.6)	143 (24.1)	81 (29.8)	256 (25.2)		
Grade12	46 (31.1)	254 (42.8)	99 (36.4)	399 (39.3)		
Family Structure					1.337	0.512
Being an only child	5 (3.4)	14 (2.4)	10 (3.7)	29 (2.9)		
Having siblings	143 (96.6)	580 (97.6)	262 (96.3)	985 (97.1)		
Class cadre					1.020	0.601
Yes	24 (16.2)	118 (19.9)	52 (19.1)	194 (19.1)		
No	124 (83.8)	476 (80.1)	219 (80.9)	820 (80.9)		

With the low-CF group as the reference group, variables with statistically significant differences in the univariate analysis were used as independent variables, and the moderate-CF group and high-CF group were used as dependent variables for multiple Logistic regression analysis. The assignment method of argument variables is shown in Table 4. The results show that female, grade11 and grade 12 are influencing factors for high cognitive fusion among high school students Hebi City, China, while female and grade 12 are independent influencing factor for the moderate-CF group, as shown in Table 5. In terms of gender, the ratio of girls entering the high-CF group is 2.147 times that of boys, and the proportion of girls entering the moderate-CF group is 1.563 times that of boys, that is, the probability of girls entering the high-CF group and moderate-CF group is significantly higher than that of boys. Grade 12 participants are 2.009 and 1.704 times more likely to be in the moderate-CF group and high-CF group, respectively. Grade 11 is 1.940 times more likely to be in the “C3 High CF Group” than Grade 10.

**Table 4:** Independent variable assignment for different potential categories potential profile factors of cognitive fusion.

Variables	Assignment method
Gender	Male = 1, Female = 2
Grade	Set the subvariables with the “Grade 10” as the reference

**Table 5:** Factors in differentiating distinct social constraints groups.

Variable	The moderate-CF group				The high-CF group			
	Coef (SE)	*P*	OR	95% CI	Coef (SE)	*P*	OR	95% CI
Gender								
Female	0.446	0.016	1.563	1.085–2.250	0.764	<0.001	2.147	1.426–3.233
Grade								
Grade11	0.467	0.052	1.595	0.995–2.557	0.663	0.012	1.940	1.156–3.256
Grade12	0.697	0.001	2.009	1.323–3.050	0.533	0.027	1.704	1.063–2.730

**Figure 2:**
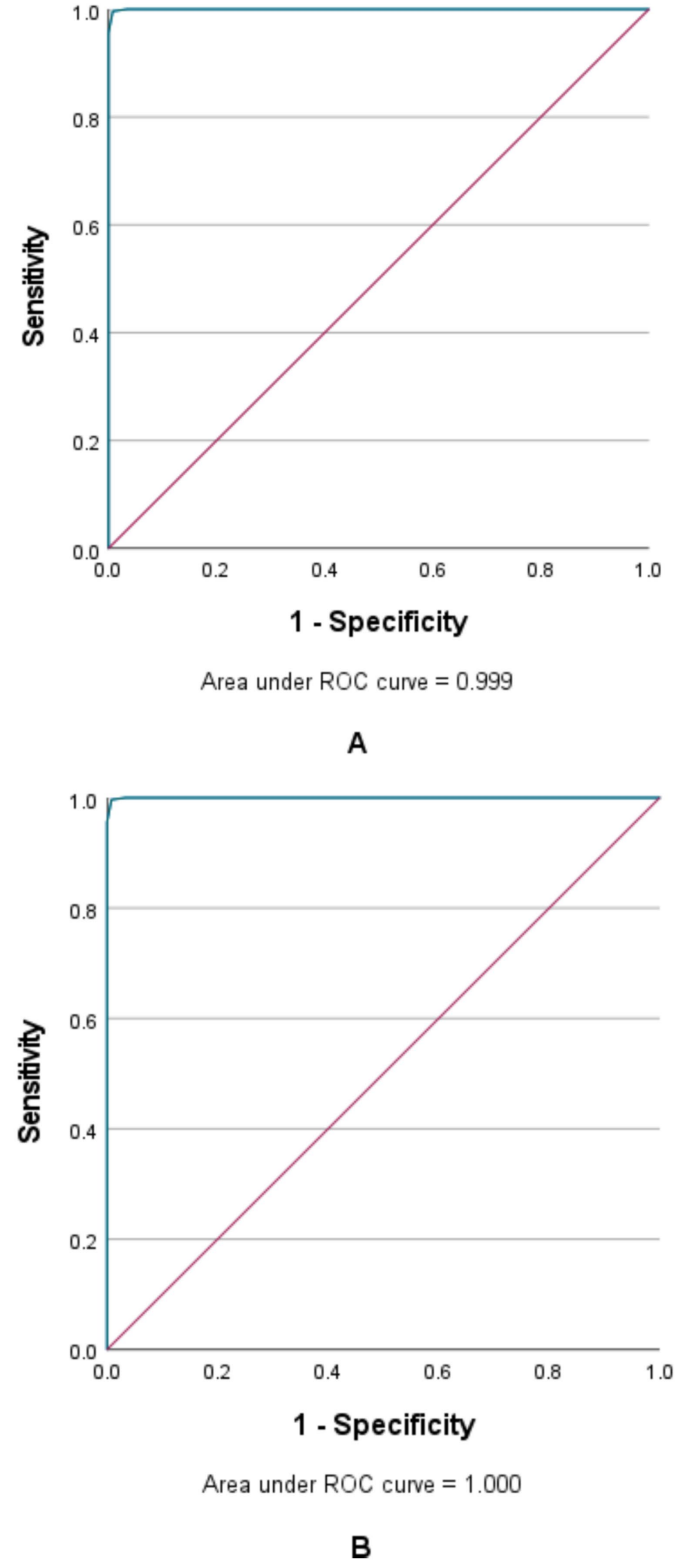
ROC curve and AUC of the predictive model. **(A)** The ROC in the high cognitive fusion group. **(B)** The ROC in the low cognitive fusion group. ROC, receiver operating characteristic; AUC, area under the curve.

According to the ROC curve, the area under ROC curve of the CFQ to distinguish the high-CF group from other CF groups was 0.999 [95%CI (0.999, 1.000)], the best cutoff value was 47.5, the sensitivity was 0.996, and the specificity was 0.992 (Figure 2A). The area under ROC curve was 1.000 [95%CI (0.999, 1.000)], the best cutoff value was 30.5, the sensitivity was 0.986, and the specificity was 0.993 (Figure 2B). When the CF score is less than 30.5, it indicates the low-CF group, when the score is between 30.5 and 47.5, it indicates the moderate-CF group, and when the score is more than 47.5, it indicates the high-CF group.

## 4 Discussion

Cognitive fusion is an important theoretical model of ACT and a critical factor in the formation and maintenance of many psychological disorders (Bohlmeijer et al., 2011; Wang et al., 2017). As evidence of the therapeutic effects of ACT confirms, CF is often associated with poorer mental health outcomes, such as depression and anxiety (Afari et al., 2019). This study aims to analyze the potential types of cognitive fusion risks among high school students, develop an effective prediction model to differentiate various risk levels, provide an empirical foundation for applying ACT in addressing psychological issues among high school students, and offer theoretical support for the targeted prevention and control of adolescent cognitive fusion as well as the more severe psychological problems that may arise from it.

### 4.1 LPA of CFQ

In this study, the CF of high school students in Hebi City, China was categorized into three distinct groups based on Zhang Weichen’s Chinese-version Creativity Factor Questionnaire (CFQ): the high-CF group, the moderate-CF group, and the low-CF group. This classification indicates the presence of heterogeneity in the CF levels among high school students. At present, there are no relevant discussions on LPA for CF. CF can significantly predict psychological distress, depression, anxiety, insomnia, hostility, academic distress, and student role problems (Barrera-Caballero et al., 2023; Krafft et al., 2019). High CF may undermine an individual’s satisfaction with their self-worth, exacerbate personal distress, and elevate stress levels. Among high school students, the proportion of those in the high-CF group is 26.8%, while the prevalence rates of anxiety and depression among Chinese high school students are 26.3 and 28.0%, respectively (Krafft et al., 2019). The similarity between these proportions and the high-CF group in this study may suggest a potential link between high cognitive fusion and psychological issues such as anxiety and depression. The moderate-CF group exhibited the highest proportion (58.6%), aligning with both expected and observed population characteristics. Although the risk of mental illness in the moderate-CF group may be lower than in the high-CF group, the large population base of this group indicates a potential for transitioning into high cognitive fusion. Therefore, parents and schools should monitor their psychological well-being closely and intervene promptly when necessary. The low-CF group represented the smallest proportion (14.5%), likely due to high school students being in a phase of rapid physical and psychological development, during which they have yet to establish a stable and positive thinking pattern. Further exploration of parenting styles and personality traits within this group could provide actionable insights for the subsequent psychological training and development of adolescents.

### 4.2 CFQ correlates

This study found females were the influencing factor of the high-CF group and moderate-CF, which was consistent with the previous studies conducted by Moreno-Montero et al. (2023) in college students and Al-Hammouri et al. (2025) in nurses. This may be related to the fact that female are more likely to fall into depression and anxiety (Sethi and Nolen-Hoeksema, 1997) and pay more attention to their own internal thinking patterns (Lisi and Staudt, 1980). It may also be attributed to the fact that girls in their mid-adolescence are in a stage of rapid psychosomatic development, where emotional instability is particularly pronounced. As a result, they often exhibit a range of contradictory behaviors, such as being both strong-willed and docile, as well as being prone to change yet also showing signs of stubbornness. But cognitive fusion, as an internalized style of cognitive assessment, may be more prevalent in female. This study identified Grade 12 as an influencing factor for both the moderate-CF group and the high-CF group, while Grade 11 was associated with the high-CF group. During these grades, students are more prone to develop negative thinking patterns and experience anxiety and depression due to increased exam pressure, a more competitive learning environment, and heightened personal and parental expectations. However, individuals in the high-CF group may display overly rigid thinking styles and severe emotional disturbances early on, which could impair their ability to engage in or complete high school studies, potentially resulting in dropout. These findings underscore the importance of not only focusing on the high-CF group but also addressing the needs of the moderate-CF group. It highlights the importance of early intervention under stressful conditions to prevent their transition into the high-CF group. This study did not find a correlation between age and cognitive fusion, and Lisi and Staudt (1980) also believed that age could not explain the cognitive development and maturity of adolescents (Hoyt et al., 2019). While this study did not identify any impact of family annual income on cognitive fusion, it also failed to review prior relevant research. Some studies have suggested that young people from high-income families exhibit healthier behavior patterns (Shao et al., 2024), but no definitive conclusion has been drawn. In addition, no significant correlation was found between residence, only child and cognitive fusion in this study.

### 4.3 CFQ’S cutoff value

According to the ROC curve analysis, a CFQ total score of < 30.5 defines the low-CF group, a score between 30.5 and 47.5 defines the moderate-CF group, and a score > 47.5 defines the high-CF group. The questionnaire demonstrates high sensitivity and specificity in identifying both the high-CF and low-CF groups. The scoring criteria established in this study can be used to judge the cognitive fusion of students in future studies. Previous studies (García-Gómez et al., 2019) have suggested that CF may be an influencing factor of depression, anxiety, PTSD and other diseases, or a manifestation of such diseases, and can also be a predictor of diseases. Early screening and intervention may improve mental flexibility and reduce the incidence of mental illness in high school students. Compared with the CFQ, depression and anxiety scales contain negative words to directly measure the clinical manifestations of depression and anxiety. The respondents might conceal the true situation due to social desirability bias, thereby failing to timely and accurately reflect the levels of depression and anxiety among the subjects (Mahard, 1988). Social expectation response refers to the fact that in a collective environment, individuals will intentionally carry out positive self-description, improve test results, and conceal their true selves to achieve a favorable situation for themselves (Gillanders et al., 2014). In this study, the 9-item CFQ primarily assesses negative thinking patterns. Its concise content helps minimize participants’ resistance to the evaluation, thereby eliciting more truthful responses and facilitating the early identification of psychological states among high school students.

### 4.4 Limitations

This study has several limitations. Firstly, all students were recruited from a single city, which might restrict the sample diversity and limit the generalizability of the findings to more extensive populations. Second, as a cross-sectional study, our results demonstrate correlations but cannot establish causal relationships. Future investigations should consider longitudinal designs to comprehensively understand the dynamic changes in cognitive fusion. Lastly, this study relied on self-reported data from students, which may introduce reporting bias.

## 5 Conclusion

Our study is pioneering in exploring cognitive fusion among Chinese high school students and has identified three distinct latent patterns. This research explores the connections between latent classes and demographic factors, including gender and grade level. The findings offer a basis for timely and effective clinical screening. This can assist parents, medical staff and teachers, identifying individuals with high levels of cognitive fusion symptoms and providing appropriate services.

## Data Availability

The raw data supporting the conclusions of this article will be made available by the authors, without undue reservation.
