# Supplementary material for: Profiles of cognitive fusion and associated factors among Chinese high school students: a latent profile analysis
Source: Front Psychol. 2025 Dec 1;16:1569773. doi: 10.3389/fpsyg.2025.1569773 (PMC12702884; doi:10.3389/fpsyg.2025.1569773)
Supplement: Supplementary file 1 [file Data_Sheet_1.zip › original data and statistical programs/Output of MPLUS with 1 classifications of CF.pdf]

```
Mplus VERSION 7.4
MUTHEN & MUTHEN
12/31/2024   8:29 PM

INPUT INSTRUCTIONS

  DATA:
    FILE IS C:\Users\dingy\Documents\认知融合.dat;
  VARIABLE:
    NAMES ARE rzrh1 rzrh2 rzrh3 rzrh4 rzrh5 rzrh6 rzrh7 rzrh8 rzrh9;

  CLASSES ARE C (1) :
   ANALYSIS:
   TYPE IS MIXTURE;
   OUTPUT:
   TECH11 TECH14;
   SAVEDATA:
   FILE IS class1.txt;
   Save IS cprob;
   PLOT:
   TYPE IS PLOT3;
   series = rzrh1-rzrh9 (*);



*** WARNING in MODEL command
  All variables are uncorrelated with all other variables within class.
  Check that this is what is intended.
*** WARNING in OUTPUT command
  TECH11 option is not available for TYPE=MIXTURE with only one class.
  Request for TECH11 is ignored.
*** WARNING in OUTPUT command
  TECH14 option is not available for TYPE=MIXTURE with only one class.
  Request for TECH14 is ignored.
   3 WARNING(S) FOUND IN THE INPUT INSTRUCTIONS




SUMMARY OF ANALYSIS

Number of groups                                                 1
Number of observations                                        1014

Number of dependent variables                                    9
Number of independent variables                                  0
Number of continuous latent variables                            0
Number of categorical latent variables                           1

Observed dependent variables

  Continuous
   RZRH1       RZRH2       RZRH3       RZRH4       RZRH5       RZRH6
   RZRH7       RZRH8       RZRH9

Categorical latent variables
   C


Estimator                                                      MLR
Information matrix                                        OBSERVED
Optimization Specifications for the Quasi-Newton Algorithm for
Continuous Outcomes
```

```
  Maximum number of iterations                                 100
  Convergence criterion                                  0.100D-05
Optimization Specifications for the EM Algorithm
  Maximum number of iterations                                 500
  Convergence criteria
    Loglikelihood change                                 0.100D-06
    Relative loglikelihood change                        0.100D-06
    Derivative                                           0.100D-05
Optimization Specifications for the M step of the EM Algorithm for
Categorical Latent variables
  Number of M step iterations                                    1
  M step convergence criterion                           0.100D-05
  Basis for M step termination                           ITERATION
Optimization Specifications for the M step of the EM Algorithm for
Censored, Binary or Ordered Categorical (Ordinal), Unordered
Categorical (Nominal) and Count Outcomes
  Number of M step iterations                                    1
  M step convergence criterion                           0.100D-05
  Basis for M step termination                           ITERATION
  Maximum value for logit thresholds                            15
  Minimum value for logit thresholds                           -15
  Minimum expected cell size for chi-square              0.100D-01
Optimization algorithm                                         EMA

Input data file(s)
  C:\Users\dingy\Documents\认知融合.dat
Input data format  FREE



THE MODEL ESTIMATION TERMINATED NORMALLY



MODEL FIT INFORMATION

Number of Free Parameters                       18

Loglikelihood

          H0 Value                      -16835.575
          H0 Scaling Correction Factor      0.9100
            for MLR

Information Criteria

          Akaike (AIC)                   33707.151
          Bayesian (BIC)                 33795.741
          Sample-Size Adjusted BIC       33738.571
            (n* = (n + 2) / 24)



FINAL CLASS COUNTS AND PROPORTIONS FOR THE LATENT CLASSES
BASED ON THE ESTIMATED MODEL

    Latent
   Classes

       1       1014.00000          1.00000


FINAL CLASS COUNTS AND PROPORTIONS FOR THE LATENT CLASSES
```

BASED ON ESTIMATED POSTERIOR PROBABILITIES

Latent Classes		
1	1014.00000	1.00000

FINAL CLASS COUNTS AND PROPORTIONS FOR THE LATENT CLASSES
BASED ON THEIR MOST LIKELY LATENT CLASS MEMBERSHIP

Class Counts and Proportions

Latent Classes		
1	1014	1.00000

Average Latent Class Probabilities for Most Likely Latent Class Membership (Row)
by Latent Class (Column)

	1
1	1.000

Classification Probabilities for the Most Likely Latent Class Membership (Column)
by Latent Class (Row)

	1
1	1.000

Logits for the Classification Probabilities for the Most Likely Latent Class Membership (Column)
by Latent Class (Row)

	1
1	0.000

MODEL RESULTS

	Estimate	S.E.	Est./S.E.	Two-Tailed P-Value
Latent Class 1				
Means				
RZRH1	4.697	0.045	104.636	0.000
RZRH2	4.469	0.047	94.776	0.000
RZRH3	4.334	0.051	84.997	0.000
RZRH4	4.570	0.049	93.382	0.000
RZRH5	4.705	0.048	97.048	0.000
RZRH6	4.687	0.047	98.724	0.000
RZRH7	4.783	0.047	101.969	0.000
RZRH8	4.281	0.048	88.779	0.000
RZRH9	4.762	0.050	95.484	0.000
Variances				
RZRH1	2.043	0.088	23.139	0.000

RZRH2	2.255	0.089	25.320	0.000
RZRH3	2.637	0.098	26.966	0.000
RZRH4	2.429	0.096	25.244	0.000
RZRH5	2.383	0.097	24.627	0.000
RZRH6	2.286	0.092	24.973	0.000
RZRH7	2.231	0.095	23.416	0.000
RZRH8	2.358	0.092	25.628	0.000
RZRH9	2.522	0.100	25.123	0.000

QUALITY OF NUMERICAL RESULTS

Condition Number for the Information Matrix 0.137E-02
(ratio of smallest to largest eigenvalue)

PLOT INFORMATION

The following plots are available:

Histograms (sample values)
Scatterplots (sample values)
Sample means
Estimated means, medians, modes, and percentiles
Sample and estimated means
Observed individual values
Estimated means and observed individual values
Estimated overall and class-specific distributions

SAVEDATA INFORMATION

Save file
class1.txt

Order and format of variables

RZRH1	F10.3
RZRH2	F10.3
RZRH3	F10.3
RZRH4	F10.3
RZRH5	F10.3
RZRH6	F10.3
RZRH7	F10.3
RZRH8	F10.3
RZRH9	F10.3
CPROB1	F10.3
C	F10.3

Save file format
11F10.3

Save file record length 10000

DIAGRAM INFORMATION

Mplus diagrams are currently not available for Mixture analysis.
No diagram output was produced.

Beginning Time: 20:29:56
Ending Time: 20:29:56

Elapsed Time: 00:00:00

MUTHEN & MUTHEN
3463 Stoner Ave.
Los Angeles, CA 90066

Tel: (310) 391-9971
Fax: (310) 391-8971
Web: www.StatModel.com
Support: Support@StatModel.com

Copyright (c) 1998-2015 Muthen & Muthen
