# Supplementary material for: Profiles of cognitive fusion and associated factors among Chinese high school students: a latent profile analysis
Source: Front Psychol. 2025 Dec 1;16:1569773. doi: 10.3389/fpsyg.2025.1569773 (PMC12702884; doi:10.3389/fpsyg.2025.1569773)
Supplement: Supplementary file 1 [file Data_Sheet_1.zip › original data and statistical programs/Output of MPLUS with 2 classifications of CF.pdf]

```
Mplus VERSION 7.4
MUTHEN & MUTHEN
12/31/2024   8:28 PM

INPUT INSTRUCTIONS

  DATA:
    FILE IS C:\Users\dingy\Documents\认知融合.dat;
  VARIABLE:
    NAMES ARE rzrh1 rzrh2 rzrh3 rzrh4 rzrh5 rzrh6 rzrh7 rzrh8 rzrh9;

  CLASSES ARE C (2) :
   ANALYSIS:
   TYPE IS MIXTURE;
   OUTPUT:
   TECH11 TECH14;
   SAVEDATA:
   FILE IS class2.txt;
   Save IS cprob;
   PLOT:
   TYPE IS PLOT3;
   series = rzrh1-rzrh9 (*);



*** WARNING in MODEL command
  All variables are uncorrelated with all other variables within class.
  Check that this is what is intended.
   1 WARNING(S) FOUND IN THE INPUT INSTRUCTIONS




SUMMARY OF ANALYSIS

Number of groups                                                 1
Number of observations                                        1014

Number of dependent variables                                    9
Number of independent variables                                  0
Number of continuous latent variables                            0
Number of categorical latent variables                           1

Observed dependent variables

  Continuous
   RZRH1       RZRH2       RZRH3       RZRH4       RZRH5       RZRH6
   RZRH7       RZRH8       RZRH9

Categorical latent variables
   C


Estimator                                                      MLR
Information matrix                                        OBSERVED
Optimization Specifications for the Quasi-Newton Algorithm for
Continuous Outcomes
  Maximum number of iterations                                 100
  Convergence criterion                                  0.100D-05
Optimization Specifications for the EM Algorithm
  Maximum number of iterations                                 500
  Convergence criteria
    Loglikelihood change                                 0.100D-06
```

```
    Relative loglikelihood change                        0.100D-06
    Derivative                                           0.100D-05
Optimization Specifications for the M step of the EM Algorithm for
Categorical Latent variables
  Number of M step iterations                                    1
  M step convergence criterion                           0.100D-05
  Basis for M step termination                           ITERATION
Optimization Specifications for the M step of the EM Algorithm for
Censored, Binary or Ordered Categorical (Ordinal), Unordered
Categorical (Nominal) and Count Outcomes
  Number of M step iterations                                    1
  M step convergence criterion                           0.100D-05
  Basis for M step termination                           ITERATION
  Maximum value for logit thresholds                            15
  Minimum value for logit thresholds                           -15
  Minimum expected cell size for chi-square              0.100D-01
Optimization algorithm                                         EMA
Random Starts Specifications
  Number of initial stage random starts                         20
  Number of final stage optimizations                            4
  Number of initial stage iterations                            10
  Initial stage convergence criterion                    0.100D+01
  Random starts scale                                    0.500D+01
  Random seed for generating random starts                       0

Input data file(s)
  C:\Users\dingy\Documents\认知融合.dat
Input data format  FREE


RANDOM STARTS RESULTS RANKED FROM THE BEST TO THE WORST LOGLIKELIHOOD VALUES

Final stage loglikelihood values at local maxima, seeds, and initial stage start numbers:

          -14903.974  637345           19
          -14903.974  285380           1
          -14903.974  415931           10
          -14903.974  68985            17



THE BEST LOGLIKELIHOOD VALUE HAS BEEN REPLICATED.  RERUN WITH AT LEAST TWICE THE
RANDOM STARTS TO CHECK THAT THE BEST LOGLIKELIHOOD IS STILL OBTAINED AND REPLICATED.


THE MODEL ESTIMATION TERMINATED NORMALLY



MODEL FIT INFORMATION

Number of Free Parameters                        28

Loglikelihood

          H0 Value                        -14903.974
          H0 Scaling Correction Factor        1.5493
            for MLR

Information Criteria

          Akaike (AIC)                     29863.948
          Bayesian (BIC)                   30001.754
```

Sample-Size Adjusted BIC 29912.824
(n* = (n + 2) / 24)

FINAL CLASS COUNTS AND PROPORTIONS FOR THE LATENT CLASSES
BASED ON THE ESTIMATED MODEL

Latent Classes		
1	572.01734	0.56412
2	441.98266	0.43588

FINAL CLASS COUNTS AND PROPORTIONS FOR THE LATENT CLASSES
BASED ON ESTIMATED POSTERIOR PROBABILITIES

Latent Classes		
1	572.01743	0.56412
2	441.98257	0.43588

FINAL CLASS COUNTS AND PROPORTIONS FOR THE LATENT CLASSES
BASED ON THEIR MOST LIKELY LATENT CLASS MEMBERSHIP

Class Counts and Proportions

Latent Classes		
1	568	0.56016
2	446	0.43984

CLASSIFICATION QUALITY

Entropy 0.876

Average Latent Class Probabilities for Most Likely Latent Class Membership (Row)
by Latent Class (Column)

	1	2
1	0.972	0.028
2	0.045	0.955

Classification Probabilities for the Most Likely Latent Class Membership (Column)
by Latent Class (Row)

	1	2
1	0.965	0.035
2	0.036	0.964

Logits for the Classification Probabilities for the Most Likely Latent Class Membership (Column)
by Latent Class (Row)

	1	2
1	3.321	0.000
2	-3.287	0.000

MODEL RESULTS

	Estimate	S.E.	Est./S.E.	Two-Tailed P-Value
**Latent Class 1**				
Means				
RZRH1	3.900	0.117	33.315	0.000
RZRH2	3.626	0.118	30.839	0.000
RZRH3	3.452	0.110	31.408	0.000
RZRH4	3.639	0.134	27.098	0.000
RZRH5	3.753	0.153	24.564	0.000
RZRH6	3.822	0.124	30.748	0.000
RZRH7	3.895	0.145	26.796	0.000
RZRH8	3.445	0.130	26.398	0.000
RZRH9	3.859	0.133	29.111	0.000
Variances				
RZRH1	1.221	0.060	20.444	0.000
RZRH2	1.333	0.061	21.692	0.000
RZRH3	1.630	0.086	18.996	0.000
RZRH4	1.308	0.059	22.273	0.000
RZRH5	1.210	0.053	22.871	0.000
RZRH6	1.316	0.064	20.500	0.000
RZRH7	1.211	0.054	22.631	0.000
RZRH8	1.452	0.065	22.385	0.000
RZRH9	1.467	0.076	19.318	0.000
**Latent Class 2**				
Means				
RZRH1	5.729	0.138	41.381	0.000
RZRH2	5.562	0.156	35.603	0.000
RZRH3	5.476	0.184	29.802	0.000
RZRH4	5.774	0.160	36.138	0.000
RZRH5	5.938	0.139	42.657	0.000
RZRH6	5.808	0.152	38.292	0.000
RZRH7	5.932	0.129	46.022	0.000
RZRH8	5.364	0.138	38.808	0.000
RZRH9	5.931	0.155	38.310	0.000
Variances				
RZRH1	1.221	0.060	20.444	0.000
RZRH2	1.333	0.061	21.692	0.000
RZRH3	1.630	0.086	18.996	0.000
RZRH4	1.308	0.059	22.273	0.000
RZRH5	1.210	0.053	22.871	0.000
RZRH6	1.316	0.064	20.500	0.000
RZRH7	1.211	0.054	22.631	0.000
RZRH8	1.452	0.065	22.385	0.000
RZRH9	1.467	0.076	19.318	0.000
**Categorical Latent Variables**				
Means				
C#1	0.258	0.228	1.129	0.259

```
QUALITY OF NUMERICAL RESULTS

     Condition Number for the Information Matrix              0.930E-02
       (ratio of smallest to largest eigenvalue)


TECHNICAL 11 OUTPUT

     Random Starts Specifications for the k-1 Class Analysis Model
        Number of initial stage random starts                  20
        Number of final stage optimizations                     4



     VUONG-LO-MENDELL-RUBIN LIKELIHOOD RATIO TEST FOR 1 (H0) VERSUS 2 CLASSES

          H0 Loglikelihood Value                        -16835.575
          2 Times the Loglikelihood Difference            3863.203
          Difference in the Number of Parameters                10
          Mean                                             438.382
          Standard Deviation                               629.968
          P-Value                                           0.0032

     LO-MENDELL-RUBIN ADJUSTED LRT TEST

          Value                                           3808.185
          P-Value                                           0.0034


TECHNICAL 14 OUTPUT

     Random Starts Specifications for the k-1 Class Analysis Model
        Number of initial stage random starts                  20
        Number of final stage optimizations                     4

     Random Starts Specification for the k-1 Class Model for Generated Data
        Number of initial stage random starts                   0
        Number of final stage optimizations for the
           initial stage random starts                          0
     Random Starts Specification for the k Class Model for Generated Data
        Number of initial stage random starts                  40
        Number of final stage optimizations                     8
     Number of bootstrap draws requested                   Varies


     PARAMETRIC BOOTSTRAPPED LIKELIHOOD RATIO TEST FOR 1 (H0) VERSUS 2 CLASSES

          H0 Loglikelihood Value                        -16835.575
          2 Times the Loglikelihood Difference            3863.203
          Difference in the Number of Parameters                10
          Approximate P-Value                               0.0000
          Successful Bootstrap Draws                             5


PLOT INFORMATION

The following plots are available:

  Histograms (sample values)
  Scatterplots (sample values)
  Sample means
```

```
  Estimated means, medians, modes, and percentiles
  Sample and estimated means
  Observed individual values
  Estimated means and observed individual values
  Estimated overall and class-specific distributions
```

SAVEDATA INFORMATION

```
  Save file
    class2.txt

  Order and format of variables

    RZRH1          F10.3
    RZRH2          F10.3
    RZRH3          F10.3
    RZRH4          F10.3
    RZRH5          F10.3
    RZRH6          F10.3
    RZRH7          F10.3
    RZRH8          F10.3
    RZRH9          F10.3
    CPROB1         F10.3
    CPROB2         F10.3
    C              F10.3

  Save file format
    12F10.3

  Save file record length    10000
```

DIAGRAM INFORMATION

```
  Mplus diagrams are currently not available for Mixture analysis.
  No diagram output was produced.


     Beginning Time:  20:28:51
        Ending Time:  20:28:52
       Elapsed Time:  00:00:01
```

MUTHEN & MUTHEN
3463 Stoner Ave.
Los Angeles, CA 90066

Tel: (310) 391-9971
Fax: (310) 391-8971
Web: www.StatModel.com
Support: Support@StatModel.com

Copyright (c) 1998-2015 Muthen & Muthen
