# Supplementary material for: Profiles of cognitive fusion and associated factors among Chinese high school students: a latent profile analysis
Source: Front Psychol. 2025 Dec 1;16:1569773. doi: 10.3389/fpsyg.2025.1569773 (PMC12702884; doi:10.3389/fpsyg.2025.1569773)
Supplement: Supplementary file 1 [file Data_Sheet_1.zip › original data and statistical programs/Output of MPLUS with 4 classifications of CF.pdf]

```
Mplus VERSION 7.4
MUTHEN & MUTHEN
12/31/2024   8:01 PM

INPUT INSTRUCTIONS

  DATA:
    FILE IS C:\Users\dingy\Documents\认知融合.dat;
  VARIABLE:
    NAMES ARE rzrh1 rzrh2 rzrh3 rzrh4 rzrh5 rzrh6 rzrh7 rzrh8 rzrh9;

  CLASSES ARE C (4) :
   ANALYSIS:
   TYPE IS MIXTURE;
   OUTPUT:
   TECH11 TECH14;
   SAVEDATA:
   FILE IS class4.txt;
   Save IS cprob;
   PLOT:
   TYPE IS PLOT3;
   series = rzrh1-rzrh9 (*);



*** WARNING in MODEL command
  All variables are uncorrelated with all other variables within class.
  Check that this is what is intended.
   1 WARNING(S) FOUND IN THE INPUT INSTRUCTIONS




SUMMARY OF ANALYSIS

Number of groups                                                 1
Number of observations                                        1014

Number of dependent variables                                    9
Number of independent variables                                  0
Number of continuous latent variables                            0
Number of categorical latent variables                           1

Observed dependent variables

  Continuous
   RZRH1       RZRH2       RZRH3       RZRH4       RZRH5       RZRH6
   RZRH7       RZRH8       RZRH9

Categorical latent variables
   C


Estimator                                                      MLR
Information matrix                                        OBSERVED
Optimization Specifications for the Quasi-Newton Algorithm for
Continuous Outcomes
  Maximum number of iterations                                 100
  Convergence criterion                                  0.100D-05
Optimization Specifications for the EM Algorithm
  Maximum number of iterations                                 500
  Convergence criteria
    Loglikelihood change                                 0.100D-06
```

```
    Relative loglikelihood change                          0.100D-06
    Derivative                                             0.100D-05
Optimization Specifications for the M step of the EM Algorithm for
Categorical Latent variables
  Number of M step iterations                                      1
  M step convergence criterion                             0.100D-05
  Basis for M step termination                             ITERATION
Optimization Specifications for the M step of the EM Algorithm for
Censored, Binary or Ordered Categorical (Ordinal), Unordered
Categorical (Nominal) and Count Outcomes
  Number of M step iterations                                      1
  M step convergence criterion                             0.100D-05
  Basis for M step termination                             ITERATION
  Maximum value for logit thresholds                              15
  Minimum value for logit thresholds                             -15
  Minimum expected cell size for chi-square                0.100D-01
Optimization algorithm                                           EMA
Random Starts Specifications
  Number of initial stage random starts                           20
  Number of final stage optimizations                              4
  Number of initial stage iterations                              10
  Initial stage convergence criterion                      0.100D+01
  Random starts scale                                      0.500D+01
  Random seed for generating random starts                         0

Input data file(s)
  C:\Users\dingy\Documents\认知融合.dat
Input data format  FREE


RANDOM STARTS RESULTS RANKED FROM THE BEST TO THE WORST LOGLIKELIHOOD VALUES

Final stage loglikelihood values at local maxima, seeds, and initial stage start numbers:

          -13209.110  76974            16
          -13209.110  637345           19
          -13209.110  68985            17
          -13209.110  573096           20



THE BEST LOGLIKELIHOOD VALUE HAS BEEN REPLICATED.  RERUN WITH AT LEAST TWICE THE
RANDOM STARTS TO CHECK THAT THE BEST LOGLIKELIHOOD IS STILL OBTAINED AND REPLICATED.


THE MODEL ESTIMATION TERMINATED NORMALLY



MODEL FIT INFORMATION

Number of Free Parameters                       48

Loglikelihood

          H0 Value                       -13209.110
          H0 Scaling Correction Factor      1.3624
            for MLR

Information Criteria

          Akaike (AIC)                    26514.220
          Bayesian (BIC)                  26750.460
```

Sample-Size Adjusted BIC 26598.008
(n* = (n + 2) / 24)

FINAL CLASS COUNTS AND PROPORTIONS FOR THE LATENT CLASSES
BASED ON THE ESTIMATED MODEL

Latent Classes		
1	360.83491	0.35585
2	341.11814	0.33641
3	108.44392	0.10695
4	203.60303	0.20079

FINAL CLASS COUNTS AND PROPORTIONS FOR THE LATENT CLASSES
BASED ON ESTIMATED POSTERIOR PROBABILITIES

Latent Classes		
1	360.83484	0.35585
2	341.11820	0.33641
3	108.44392	0.10695
4	203.60303	0.20079

FINAL CLASS COUNTS AND PROPORTIONS FOR THE LATENT CLASSES
BASED ON THEIR MOST LIKELY LATENT CLASS MEMBERSHIP

Class Counts and Proportions

Latent Classes		
1	371	0.36588
2	344	0.33925
3	103	0.10158
4	196	0.19329

CLASSIFICATION QUALITY

Entropy 0.896

Average Latent Class Probabilities for Most Likely Latent Class Membership (Row)
by Latent Class (Column)

	1	2	3	4
1	0.923	0.059	0.017	0.000
2	0.050	0.919	0.000	0.031
3	0.010	0.000	0.990	0.000
4	0.000	0.015	0.000	0.985

Classification Probabilities for the Most Likely Latent Class Membership (Column)
by Latent Class (Row)

	1	2	3	4

1	0.949	0.048	0.003	0.000
2	0.064	0.927	0.000	0.009
3	0.060	0.000	0.940	0.000
4	0.000	0.052	0.000	0.948

Logits for the Classification Probabilities for the Most Likely Latent Class Membership (Column)
by Latent Class (Row)

	1	2	3	4
1	13.764	10.775	7.967	0.000
2	1.981	4.648	-9.092	0.000
3	10.999	0.000	13.754	0.000
4	-13.762	-2.899	-13.762	0.000

MODEL RESULTS

	Estimate	S.E.	Est./S.E.	Two-Tailed P-Value
**Latent Class 1**				
Means				
RZRH1	4.119	0.076	54.060	0.000
RZRH2	3.868	0.075	51.702	0.000
RZRH3	3.655	0.087	41.792	0.000
RZRH4	3.900	0.092	42.276	0.000
RZRH5	3.958	0.112	35.388	0.000
RZRH6	3.996	0.097	41.334	0.000
RZRH7	4.107	0.091	45.319	0.000
RZRH8	3.653	0.088	41.563	0.000
RZRH9	4.102	0.095	43.039	0.000
Variances				
RZRH1	0.824	0.048	17.207	0.000
RZRH2	0.850	0.060	14.273	0.000
RZRH3	1.146	0.069	16.649	0.000
RZRH4	0.697	0.054	12.951	0.000
RZRH5	0.562	0.042	13.275	0.000
RZRH6	0.853	0.060	14.128	0.000
RZRH7	0.642	0.045	14.195	0.000
RZRH8	1.013	0.068	14.983	0.000
RZRH9	0.958	0.075	12.814	0.000
**Latent Class 2**				
Means				
RZRH1	5.002	0.095	52.916	0.000
RZRH2	4.710	0.087	54.393	0.000
RZRH3	4.563	0.115	39.519	0.000
RZRH4	4.970	0.110	45.348	0.000
RZRH5	5.211	0.095	54.777	0.000
RZRH6	5.078	0.099	51.227	0.000
RZRH7	5.268	0.098	54.013	0.000
RZRH8	4.615	0.088	52.307	0.000
RZRH9	5.168	0.111	46.463	0.000
Variances				
RZRH1	0.824	0.048	17.207	0.000
RZRH2	0.850	0.060	14.273	0.000

RZRH3	1.146	0.069	16.649	0.000
RZRH4	0.697	0.054	12.951	0.000
RZRH5	0.562	0.042	13.275	0.000
RZRH6	0.853	0.060	14.128	0.000
RZRH7	0.642	0.045	14.195	0.000
RZRH8	1.013	0.068	14.983	0.000
RZRH9	0.958	0.075	12.814	0.000

Latent Class 3

Means

RZRH1	2.521	0.152	16.605	0.000
RZRH2	2.197	0.147	14.954	0.000
RZRH3	2.142	0.134	16.035	0.000
RZRH4	1.901	0.135	14.080	0.000
RZRH5	1.984	0.139	14.248	0.000
RZRH6	2.391	0.154	15.532	0.000
RZRH7	2.188	0.179	12.250	0.000
RZRH8	2.020	0.115	17.604	0.000
RZRH9	2.241	0.152	14.726	0.000

Variances

RZRH1	0.824	0.048	17.207	0.000
RZRH2	0.850	0.060	14.273	0.000
RZRH3	1.146	0.069	16.649	0.000
RZRH4	0.697	0.054	12.951	0.000
RZRH5	0.562	0.042	13.275	0.000
RZRH6	0.853	0.060	14.128	0.000
RZRH7	0.642	0.045	14.195	0.000
RZRH8	1.013	0.068	14.983	0.000
RZRH9	0.958	0.075	12.814	0.000

Latent Class 4

Means

RZRH1	6.371	0.077	82.920	0.000
RZRH2	6.343	0.112	56.399	0.000
RZRH3	6.324	0.094	66.983	0.000
RZRH4	6.510	0.069	93.738	0.000
RZRH5	6.631	0.057	116.487	0.000
RZRH6	6.482	0.071	91.701	0.000
RZRH7	6.551	0.061	107.763	0.000
RZRH8	6.039	0.125	48.429	0.000
RZRH9	6.595	0.072	92.226	0.000

Variances

RZRH1	0.824	0.048	17.207	0.000
RZRH2	0.850	0.060	14.273	0.000
RZRH3	1.146	0.069	16.649	0.000
RZRH4	0.697	0.054	12.951	0.000
RZRH5	0.562	0.042	13.275	0.000
RZRH6	0.853	0.060	14.128	0.000
RZRH7	0.642	0.045	14.195	0.000
RZRH8	1.013	0.068	14.983	0.000
RZRH9	0.958	0.075	12.814	0.000

Categorical Latent Variables

Means

C#1	0.572	0.160	3.587	0.000
C#2	0.516	0.105	4.925	0.000
C#3	-0.630	0.186	-3.395	0.001

```
QUALITY OF NUMERICAL RESULTS

     Condition Number for the Information Matrix              0.498E-02
       (ratio of smallest to largest eigenvalue)


TECHNICAL 11 OUTPUT

     Random Starts Specifications for the k-1 Class Analysis Model
        Number of initial stage random starts                  20
        Number of final stage optimizations                     4



     VUONG-LO-MENDELL-RUBIN LIKELIHOOD RATIO TEST FOR 3 (H0) VERSUS 4 CLASSES

          H0 Loglikelihood Value                       -13550.024
          2 Times the Loglikelihood Difference            681.828
          Difference in the Number of Parameters               10
          Mean                                             62.326
          Standard Deviation                              142.835
          P-Value                                          0.0041

     LO-MENDELL-RUBIN ADJUSTED LRT TEST

          Value                                           672.117
          P-Value                                          0.0044


TECHNICAL 14 OUTPUT

     Random Starts Specifications for the k-1 Class Analysis Model
        Number of initial stage random starts                  20
        Number of final stage optimizations                     4

     Random Starts Specification for the k-1 Class Model for Generated Data
        Number of initial stage random starts                   0
        Number of final stage optimizations for the
           initial stage random starts                          0
     Random Starts Specification for the k Class Model for Generated Data
        Number of initial stage random starts                  40
        Number of final stage optimizations                     8
     Number of bootstrap draws requested                   Varies


     PARAMETRIC BOOTSTRAPPED LIKELIHOOD RATIO TEST FOR 3 (H0) VERSUS 4 CLASSES

          H0 Loglikelihood Value                       -13550.024
          2 Times the Loglikelihood Difference            681.828
          Difference in the Number of Parameters               10
          Approximate P-Value                              0.0000
          Successful Bootstrap Draws                            5


PLOT INFORMATION

The following plots are available:

  Histograms (sample values)
  Scatterplots (sample values)
  Sample means
  Estimated means, medians, modes, and percentiles
```

```
  Sample and estimated means
  Observed individual values
  Estimated means and observed individual values
  Estimated overall and class-specific distributions

SAVEDATA INFORMATION


  Save file
    class4.txt

  Order and format of variables

    RZRH1          F10.3
    RZRH2          F10.3
    RZRH3          F10.3
    RZRH4          F10.3
    RZRH5          F10.3
    RZRH6          F10.3
    RZRH7          F10.3
    RZRH8          F10.3
    RZRH9          F10.3
    CPROB1         F10.3
    CPROB2         F10.3
    CPROB3         F10.3
    CPROB4         F10.3
    C              F10.3

  Save file format
    14F10.3

  Save file record length    10000


DIAGRAM INFORMATION

  Mplus diagrams are currently not available for Mixture analysis.
  No diagram output was produced.


     Beginning Time:  20:01:52
        Ending Time:  20:01:54
       Elapsed Time:  00:00:02
```

MUTHEN & MUTHEN
3463 Stoner Ave.
Los Angeles, CA 90066

Tel: (310) 391-9971
Fax: (310) 391-8971
Web: www.StatModel.com
Support: Support@StatModel.com

Copyright (c) 1998-2015 Muthen & Muthen
