# Supplementary material for: Profiles of cognitive fusion and associated factors among Chinese high school students: a latent profile analysis
Source: Front Psychol. 2025 Dec 1;16:1569773. doi: 10.3389/fpsyg.2025.1569773 (PMC12702884; doi:10.3389/fpsyg.2025.1569773)
Supplement: Supplementary file 1 [file Data_Sheet_1.zip › original data and statistical programs/Output of MPLUS with 5 classifications of CF.pdf]

```
Mplus VERSION 7.4
MUTHEN & MUTHEN
12/31/2024   7:42 PM

INPUT INSTRUCTIONS

  DATA:
    FILE IS C:\Users\dingy\Documents\认知融合.dat;
  VARIABLE:
    NAMES ARE rzrh1 rzrh2 rzrh3 rzrh4 rzrh5 rzrh6 rzrh7 rzrh8 rzrh9;

  CLASSES ARE C (5) :
   ANALYSIS:
   TYPE IS MIXTURE;
   OUTPUT:
   TECH11 TECH14;
   SAVEDATA:
   FILE IS class5.txt;
   Save IS cprob;
   PLOT:
   TYPE IS PLOT3;
   series = rzrh1-rzrh9 (*);



*** WARNING in MODEL command
  All variables are uncorrelated with all other variables within class.
  Check that this is what is intended.
   1 WARNING(S) FOUND IN THE INPUT INSTRUCTIONS




SUMMARY OF ANALYSIS

Number of groups                                                 1
Number of observations                                        1014

Number of dependent variables                                    9
Number of independent variables                                  0
Number of continuous latent variables                            0
Number of categorical latent variables                           1

Observed dependent variables

  Continuous
   RZRH1       RZRH2       RZRH3       RZRH4       RZRH5       RZRH6
   RZRH7       RZRH8       RZRH9

Categorical latent variables
   C


Estimator                                                      MLR
Information matrix                                        OBSERVED
Optimization Specifications for the Quasi-Newton Algorithm for
Continuous Outcomes
  Maximum number of iterations                                 100
  Convergence criterion                                  0.100D-05
Optimization Specifications for the EM Algorithm
  Maximum number of iterations                                 500
  Convergence criteria
    Loglikelihood change                                 0.100D-06
```

```
    Relative loglikelihood change                          0.100D-06
    Derivative                                             0.100D-05
Optimization Specifications for the M step of the EM Algorithm for
Categorical Latent variables
  Number of M step iterations                                      1
  M step convergence criterion                             0.100D-05
  Basis for M step termination                             ITERATION
Optimization Specifications for the M step of the EM Algorithm for
Censored, Binary or Ordered Categorical (Ordinal), Unordered
Categorical (Nominal) and Count Outcomes
  Number of M step iterations                                      1
  M step convergence criterion                             0.100D-05
  Basis for M step termination                             ITERATION
  Maximum value for logit thresholds                              15
  Minimum value for logit thresholds                             -15
  Minimum expected cell size for chi-square                0.100D-01
Optimization algorithm                                           EMA
Random Starts Specifications
  Number of initial stage random starts                           20
  Number of final stage optimizations                              4
  Number of initial stage iterations                              10
  Initial stage convergence criterion                      0.100D+01
  Random starts scale                                      0.500D+01
  Random seed for generating random starts                         0

Input data file(s)
  C:\Users\dingy\Documents\认知融合.dat
Input data format  FREE


RANDOM STARTS RESULTS RANKED FROM THE BEST TO THE WORST LOGLIKELIHOOD VALUES

Final stage loglikelihood values at local maxima, seeds, and initial stage start numbers:

          -13066.646  127215           9
          -13066.646  68985            17
          -13074.257  939021           8
          -13074.257  93468            3



THE BEST LOGLIKELIHOOD VALUE HAS BEEN REPLICATED.  RERUN WITH AT LEAST TWICE THE
RANDOM STARTS TO CHECK THAT THE BEST LOGLIKELIHOOD IS STILL OBTAINED AND REPLICATED.


THE MODEL ESTIMATION TERMINATED NORMALLY



MODEL FIT INFORMATION

Number of Free Parameters                        58

Loglikelihood

          H0 Value                       -13066.646
          H0 Scaling Correction Factor       1.3117
            for MLR

Information Criteria

          Akaike (AIC)                    26249.291
          Bayesian (BIC)                  26534.747
```

Sample-Size Adjusted BIC 26350.535
(n* = (n + 2) / 24)

FINAL CLASS COUNTS AND PROPORTIONS FOR THE LATENT CLASSES
BASED ON THE ESTIMATED MODEL

Latent Classes		
1	50.61706	0.04992
2	107.81184	0.10632
3	293.49573	0.28944
4	369.51249	0.36441
5	192.56288	0.18990

FINAL CLASS COUNTS AND PROPORTIONS FOR THE LATENT CLASSES
BASED ON ESTIMATED POSTERIOR PROBABILITIES

Latent Classes		
1	50.61706	0.04992
2	107.81183	0.10632
3	293.49571	0.28944
4	369.51251	0.36441
5	192.56288	0.18990

FINAL CLASS COUNTS AND PROPORTIONS FOR THE LATENT CLASSES
BASED ON THEIR MOST LIKELY LATENT CLASS MEMBERSHIP

Class Counts and Proportions

Latent Classes		
1	49	0.04832
2	109	0.10750
3	297	0.29290
4	367	0.36193
5	192	0.18935

CLASSIFICATION QUALITY

Entropy 0.895

Average Latent Class Probabilities for Most Likely Latent Class Membership (Row)
by Latent Class (Column)

	1	2	3	4	5
1	0.961	0.039	0.000	0.000	0.000
2	0.032	0.920	0.000	0.048	0.000
3	0.000	0.000	0.908	0.074	0.019
4	0.000	0.015	0.052	0.933	0.000
5	0.000	0.000	0.026	0.000	0.974

Classification Probabilities for the Most Likely Latent Class Membership (Column)
by Latent Class (Row)

	1	2	3	4	5
1	0.931	0.069	0.000	0.000	0.000
2	0.018	0.930	0.000	0.053	0.000
3	0.000	0.000	0.918	0.064	0.017
4	0.000	0.014	0.059	0.927	0.000
5	0.000	0.000	0.029	0.000	0.971

Logits for the Classification Probabilities for the Most Likely Latent Class Membership (Column)
by Latent Class (Row)

	1	2	3	4	5
1	13.744	11.146	0.000	0.000	0.000
2	9.773	13.743	0.000	10.870	0.000
3	-9.752	-9.752	3.978	1.321	0.000
4	0.000	9.559	10.987	13.739	0.000
5	-13.786	-13.786	-3.506	-13.786	0.000

MODEL RESULTS

	Estimate	S.E.	Est./S.E.	Two-Tailed P-Value
**Latent Class 1**				
Means				
RZRH1	1.751	0.213	8.227	0.000
RZRH2	1.697	0.191	8.864	0.000
RZRH3	1.721	0.142	12.139	0.000
RZRH4	1.493	0.131	11.433	0.000
RZRH5	1.565	0.171	9.158	0.000
RZRH6	1.812	0.229	7.923	0.000
RZRH7	1.712	0.257	6.660	0.000
RZRH8	1.562	0.188	8.294	0.000
RZRH9	1.781	0.232	7.667	0.000
Variances				
RZRH1	0.763	0.048	15.932	0.000
RZRH2	0.797	0.053	15.147	0.000
RZRH3	1.120	0.071	15.876	0.000
RZRH4	0.659	0.054	12.175	0.000
RZRH5	0.524	0.038	13.933	0.000
RZRH6	0.802	0.060	13.351	0.000
RZRH7	0.614	0.043	14.201	0.000
RZRH8	0.947	0.058	16.263	0.000
RZRH9	0.920	0.074	12.445	0.000
**Latent Class 2**				
Means				
RZRH1	3.444	0.197	17.440	0.000
RZRH2	2.927	0.151	19.319	0.000
RZRH3	2.771	0.178	15.546	0.000
RZRH4	2.644	0.211	12.506	0.000
RZRH5	2.684	0.156	17.206	0.000
RZRH6	3.091	0.174	17.775	0.000
RZRH7	2.962	0.171	17.337	0.000

```
    RZRH8              2.629      0.143     18.418      0.000
    RZRH9              2.976      0.199     14.925      0.000

 Variances
    RZRH1              0.763      0.048     15.932      0.000
    RZRH2              0.797      0.053     15.147      0.000
    RZRH3              1.120      0.071     15.876      0.000
    RZRH4              0.659      0.054     12.175      0.000
    RZRH5              0.524      0.038     13.933      0.000
    RZRH6              0.802      0.060     13.351      0.000
    RZRH7              0.614      0.043     14.201      0.000
    RZRH8              0.947      0.058     16.263      0.000
    RZRH9              0.920      0.074     12.445      0.000

Latent Class 3

 Means
    RZRH1              5.115      0.082     62.622      0.000
    RZRH2              4.818      0.083     57.816      0.000
    RZRH3              4.679      0.101     46.413      0.000
    RZRH4              5.101      0.097     52.561      0.000
    RZRH5              5.331      0.074     72.139      0.000
    RZRH6              5.197      0.084     61.936      0.000
    RZRH7              5.379      0.071     75.398      0.000
    RZRH8              4.712      0.074     63.367      0.000
    RZRH9              5.295      0.093     56.735      0.000

 Variances
    RZRH1              0.763      0.048     15.932      0.000
    RZRH2              0.797      0.053     15.147      0.000
    RZRH3              1.120      0.071     15.876      0.000
    RZRH4              0.659      0.054     12.175      0.000
    RZRH5              0.524      0.038     13.933      0.000
    RZRH6              0.802      0.060     13.351      0.000
    RZRH7              0.614      0.043     14.201      0.000
    RZRH8              0.947      0.058     16.263      0.000
    RZRH9              0.920      0.074     12.445      0.000

Latent Class 4

 Means
    RZRH1              4.244      0.060     70.668      0.000
    RZRH2              4.014      0.066     60.501      0.000
    RZRH3              3.816      0.079     48.532      0.000
    RZRH4              4.102      0.065     63.090      0.000
    RZRH5              4.207      0.089     47.219      0.000
    RZRH6              4.190      0.074     56.691      0.000
    RZRH7              4.324      0.079     54.984      0.000
    RZRH8              3.845      0.076     50.273      0.000
    RZRH9              4.298      0.074     58.173      0.000

 Variances
    RZRH1              0.763      0.048     15.932      0.000
    RZRH2              0.797      0.053     15.147      0.000
    RZRH3              1.120      0.071     15.876      0.000
    RZRH4              0.659      0.054     12.175      0.000
    RZRH5              0.524      0.038     13.933      0.000
    RZRH6              0.802      0.060     13.351      0.000
    RZRH7              0.614      0.043     14.201      0.000
    RZRH8              0.947      0.058     16.263      0.000
    RZRH9              0.920      0.074     12.445      0.000

Latent Class 5
```

Means

RZRH1	6.406	0.064	100.278	0.000
RZRH2	6.404	0.077	83.333	0.000
RZRH3	6.366	0.081	78.144	0.000
RZRH4	6.545	0.060	108.684	0.000
RZRH5	6.664	0.046	143.761	0.000
RZRH6	6.515	0.060	109.162	0.000
RZRH7	6.581	0.053	124.066	0.000
RZRH8	6.100	0.094	65.174	0.000
RZRH9	6.625	0.061	108.716	0.000

Variances

RZRH1	0.763	0.048	15.932	0.000
RZRH2	0.797	0.053	15.147	0.000
RZRH3	1.120	0.071	15.876	0.000
RZRH4	0.659	0.054	12.175	0.000
RZRH5	0.524	0.038	13.933	0.000
RZRH6	0.802	0.060	13.351	0.000
RZRH7	0.614	0.043	14.201	0.000
RZRH8	0.947	0.058	16.263	0.000
RZRH9	0.920	0.074	12.445	0.000

Categorical Latent Variables

Means

C#1	-1.336	0.277	-4.826	0.000
C#2	-0.580	0.146	-3.981	0.000
C#3	0.421	0.108	3.894	0.000
C#4	0.652	0.122	5.354	0.000

QUALITY OF NUMERICAL RESULTS

Condition Number for the Information Matrix 0.325E-03
(ratio of smallest to largest eigenvalue)

TECHNICAL 11 OUTPUT

Random Starts Specifications for the k-1 Class Analysis Model

Number of initial stage random starts	20
Number of final stage optimizations	4

VUONG-LO-MENDELL-RUBIN LIKELIHOOD RATIO TEST FOR 4 (H0) VERSUS 5 CLASSES

H0 Loglikelihood Value	-13209.110
2 Times the Loglikelihood Difference	284.929
Difference in the Number of Parameters	10
Mean	-12.485
Standard Deviation	145.923
P-Value	0.0167

LO-MENDELL-RUBIN ADJUSTED LRT TEST

Value	280.871
P-Value	0.0174

TECHNICAL 14 OUTPUT

```
     Random Starts Specifications for the k-1 Class Analysis Model
        Number of initial stage random starts                  20
        Number of final stage optimizations                     4

     Random Starts Specification for the k-1 Class Model for Generated Data
        Number of initial stage random starts                   0
        Number of final stage optimizations for the
           initial stage random starts                          0
     Random Starts Specification for the k Class Model for Generated Data
        Number of initial stage random starts                  40
        Number of final stage optimizations                     8
     Number of bootstrap draws requested                   Varies


     PARAMETRIC BOOTSTRAPPED LIKELIHOOD RATIO TEST FOR 4 (H0) VERSUS 5 CLASSES

          H0 Loglikelihood Value                       -13209.110
          2 Times the Loglikelihood Difference             284.929
          Difference in the Number of Parameters                10
          Approximate P-Value                               0.0000
          Successful Bootstrap Draws                             5


PLOT INFORMATION

The following plots are available:

  Histograms (sample values)
  Scatterplots (sample values)
  Sample means
  Estimated means, medians, modes, and percentiles
  Sample and estimated means
  Observed individual values
  Estimated means and observed individual values
  Estimated overall and class-specific distributions

SAVEDATA INFORMATION


  Save file
    class5.txt

  Order and format of variables

    RZRH1          F10.3
    RZRH2          F10.3
    RZRH3          F10.3
    RZRH4          F10.3
    RZRH5          F10.3
    RZRH6          F10.3
    RZRH7          F10.3
    RZRH8          F10.3
    RZRH9          F10.3
    CPROB1         F10.3
    CPROB2         F10.3
    CPROB3         F10.3
    CPROB4         F10.3
    CPROB5         F10.3
    C              F10.3

  Save file format
    15F10.3
```

```
  Save file record length    10000


DIAGRAM INFORMATION

  Mplus diagrams are currently not available for Mixture analysis.
  No diagram output was produced.


     Beginning Time:  19:42:44
        Ending Time:  19:42:47
       Elapsed Time:  00:00:03
```

MUTHEN & MUTHEN
3463 Stoner Ave.
Los Angeles, CA 90066

Tel: (310) 391-9971
Fax: (310) 391-8971
Web: www.StatModel.com
Support: Support@StatModel.com

Copyright (c) 1998-2015 Muthen & Muthen
